# The 2026 ISCB Accomplishments by a Senior Scientist Award—Dr Richard Durbin

**DOI:** 10.1093/bioinformatics/btag283

**Published:** 2026-07-07

**Authors:** Mallory L Wiper

**Affiliations:** The International Society for Computational Biology, 525K East Market Street, RM 330, Leesburg, VA 20176, United States


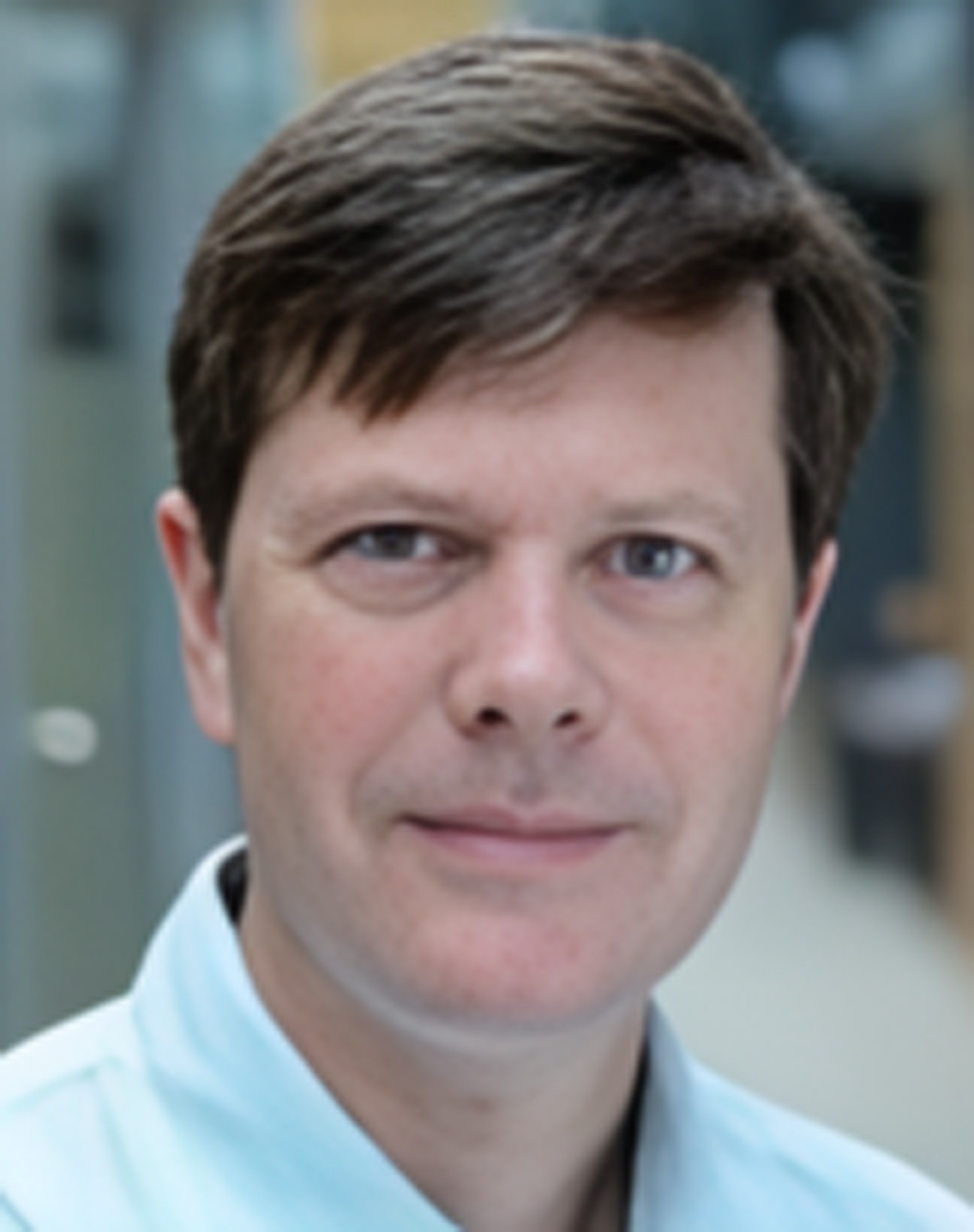


This year, at the 34th Intelligent Systems for Molecular Biology (ISMB) conference in Washington, D.C., the International Society for Computational Biology (ISCB) is proud to present the 2026 Accomplishments by a Senior Scientist Award to Dr Richard Durbin in recognition of his role in shaping large-scale analysis of genetic variation and the development of foundational methods for genome sequencing and evolutionary genomics.

## 1 Math, evolution, and computational biology

Dr Durbin describes his path to computational biology as having “very mathematical beginnings.” His early aptitude for math was evident in high school, where a teacher recognized Durbin’s mathematical talent and pushed him beyond the standard curriculum, eventually leading him to compete at both the national and international levels of the Math Olympiad. At home, his intellectual sparring with his father further sharpened his thinking. Those debates, he recalls, made him “fiercely logical” and shaped the way he approached problems.

Yet fierce logic and an aptitude for mathematics alone would not define his academic trajectory. Between high school and university, Durbin spent a year working for a computer consultancy, where he first learned to program—an introduction to computation that would prove pivotal later in his career. After his year with the consultancy learning programming and computational skills, Durbin began his undergraduate studies. He arrived at Cambridge excited to pursue his degree in mathematics, but as the degree progressed, the focus felt too narrow and, for Durbin had become “a combination of harder and more boring.” With a wish to move away from math, it was his brother’s scientific interests that influenced his next steps.

Durbin remembers his younger brother’s curiosity about biology and evolution as being an influential force on the family, piquing his own interest in the evolution of life on Earth, a course of study he now hoped to pursue. Having given up any formal study of biology at a young age, however, Durbin anticipated obstacles in getting into graduate programs focused on biology. Nevertheless, he persisted in searching for ways to study biology more formally. That opportunity came when he received a Fulbright Award, which allowed him to spend a year at Harvard in the biophysics program, studying under Wally Gilbert. Because this program didn’t require a biology GRE, Durbin was able to use his biophysics studies as the transition into biology he was looking for.

After Harvard, Durbin was offered a PhD position in Cambridge at the MRC Laboratory of Molecular Biology, where he worked on modeling the nervous system of *Caenorhabditis elegans*. His work with *C. elegans* led to an interest in neural network models, which he was able to explore further during his postdoc at Stanford with David Rumelhart, a founder of backpropagation and early machine learning. Soon after, Durbin received an invitation from John Sulston at the Sanger Institute to help with founding the Human Genome Project. He accepted, joining the effort to handle its computational work.

Looking back on what he referred to as his meandering academic career path—including this crucial entry point into what would be an important part of his bioinformatics career—Durbin said that he’s been fortunate to have been in the right place at the right time to take advantage of the opportunities that came his way.

## 2 Mentors and influences

Reflecting on influential mentors, Durbin named John Sulston as the most important figure in his career. Sulston was hugely influential in shaping the landscape of computational biology in Europe through establishing the Sanger Institute and positioning the European Bioinformatics Institute on the same campus. Most notably for Durbin, Sulston brought Durbin into the early genome sequencing effort at Sanger, placing him at the center of what would become the Human Genome Project.

Other mentors in Durbin’s career included Steve Harrison and Don Wiley, structural biologists he worked with during his time at Harvard, as well as David Rumelhart, whom he worked with at Stanford during his postdoc researching neural modeling. Another notable mentor was Sydney Brenner. Though Durbin never worked directly with him, Brenner was a towering figure in computational biology in the late 20th century whose work was very influential on Durbin’s own research.

Finally, Durbin said his students have been, and continue to be, key mentors in his research. Naming Ewan Birney and Heng Li, among others, he said that he’s always learning from his students, a reminder that scientific growth and progress are rarely one-sided but are a collective effort.

## 3 Mentoring the next generation

When asked about his approach to mentorship, Durbin described his mentoring style jokingly as “constructive neglect.” He recalled rebelling against the direction of his own PhD advisor and ultimately benefiting from the freedom to follow his own interests. That experience shaped how he supervises his own students: allowing them the space to pursue their interests and talents and to develop independence as researchers. As he put it, “studentship is for the student, not the PI.”

Durbin also puts into practice his belief that doctoral training should be completed as quickly and efficiently as possible, allowing young scientists to move on to the next stage of their careers. He compares the academic path to a medieval guild system: PhD students are apprentices, post-docs are journeymen, and independent scientists are master craftspeople. In this view, the goal is to move through these stages efficiently, earning the qualification, and then deciding where that training may lead.

## 4 Research methods

Durbin said that his values surrounding the pursuit of science haven’t changed from what they were during his time at the MRC Laboratory of Molecular Biology. A core principle instilled in him is summarized most accurately by a quote from Max Perutz that was displayed outside the lecture theater at LMB: “In science, truth always wins.” For Durbin, science continues to be about establishing truth.

He contrasted this idea with the current focus in science on journal placement and publication metrics. Though he acknowledged the importance of publications in sharing discoveries and new findings, Durbin’s stance is that success shouldn’t be measured solely by the number of publications but also by the influence and broader-reaching impact of one’s work.

## 5 Findings and future focus

A major finding that shaped much of Durbin’s research was a product of the Human Genome Project. Specifically, the finding that the human genome contains only 20 000 protein-coding genes instead of the 50 000–100 000 genes that many had predicted. With this surprising finding and his deep involvement in gene identification as part of this project, Durbin’s attention shifted toward gene regulation.

Durbin has also pushed for the sequencing of many human genomes, advocating for low-coverage population sequencing and what became the Thousand Genomes Project. He has also been instrumental in advancing imputation techniques, allowing the transfer of information from fully sequenced genomes to genotyped individuals.

Through his work with genome projects and sequencing techniques, Durbin noted that human genetics is fundamentally different than other organisms because of how outbred we are and how widely our genomes vary. Thus, Durbin’s focus moved to evolutionary and population genetics, developing methods to analyze genetic variation to better understand our (and other species’) evolutionary past.

Looking ahead, Durbin is continuing his sequencing pursuits, working on producing a reference genome for all species, which he’s stated is “just as transformative as having the human genome.” Current opposition to this pursuit mirrors sequencing skepticism from the 1990s, but he argues that having complete reference genomes will only benefit the study of biology, allowing systematic testing, mapping, and interpretation.

His aspiration would be for the barrier between molecular genomics and organismal ecology to break down, leading to what he sees as the truly unified study of evolution. A complete genomic reference for life, Durbin argues, would help bridge this gap.

## 6 Reflections on the Accomplishments by a Senior Scientist Award

When asked how he feels being the 2026 winner of the ISCB Accomplishments by a Senior Scientist Award, Durbin said that he was deeply honored and a bit amazed. He expressed his gratitude to the Society and his pride at being named among previous winners of the award, many of whom are respected peers.

In reflecting on the award, Durbin said he’s excited for what’s next in the study of evolutionary genomics and that he’s still writing code and continuing to develop computational methods to make genomic analysis faster and more efficient.

